# Supplementary material for: Longitudinal performance of the ENLIST ENL severity scale in individuals with severe erythema nodosum leprosum: Responsiveness, trajectories and clinical features – a secondary analysis of the Methotrexate and Prednisolone study - MaPs in ENL
Source: PLoS Negl Trop Dis. 2026 Aug 3;20(8):e0014410. doi: 10.1371/journal.pntd.0014410 (PMC13460738; doi:10.1371/journal.pntd.0014410)
Supplement: Supplementary file 1 [file pntd.0014410.s001.pdf]

CONSORT checklist (adapted for secondary/post-hoc analysis)

CONSORT Item	Description	Reported in manuscript
**1a**	Identification as a randomised trial	Title and abstract
**1b**	Structured summary of trial design, methods, results and conclusions	Abstract
**2**	Trial registration	Methods/Title page
**3**	Protocol availability	Supplementary protocol file
**4**	Data sharing statement	Data availability statement
**5a**	Funding	Title page/Funding statement
**5b**	Conflicts of interest	Title page/Conflicts statement
**6**	Scientific background and rationale	Introduction
**7**	Objectives related to EESS outcomes	Introduction/Objectives
**8**	Patient/public involvement	Not applicable
**9**	Trial design	Methods
**10**	Changes to protocol	Not applicable to this secondary analysis
**11**	Trial setting and locations	Methods
**12a**	Participant eligibility criteria	Methods
**13**	Intervention and comparator	Reported in primary trial publication and briefly summarised in Methods
**14**	Outcomes analysed in this secondary analysis (EESS)	Methods
**15**	Harms assessment	Not applicable to this analysis
**16a**	Sample size	Sample size determined in primary trial publication
**16b**	Interim analyses/stopping guidelines	Not applicable
**17a–20b**	Randomisation, allocation concealment and blinding	Reported in primary MaPs in ENL trial publication
**21a**	Statistical methods	Statistical analysis section
**21b**	Definition of analysed population	Statistical analysis section
**21c**	Missing data handling	Statistical analysis section
**21d**	Additional analyses	Statistical analysis section
**22a–22b**	Participant flow and analysed population	Results
**23a**	Recruitment and follow-up dates	Methods

**23b**	Trial stopping	Not applicable
**24a–24b**	Intervention delivery/concomitant care	Reported in primary trial publication
**25**	Baseline characteristics	Table 1
**26**	Numbers analysed and effect estimates	Results/Table 1
**27**	Harms	Not applicable to this analysis
**28**	Ancillary analyses	Results
**29**	Interpretation	Discussion
**30**	Limitations	Discussion
